# Alteration in Taste Perception in Cancer: Causes and Strategies of Treatment

**DOI:** 10.3389/fphys.2017.00134

**Published:** 2017-03-08

**Authors:** Babar Murtaza, Aziz Hichami, Amira S. Khan, François Ghiringhelli, Naim A. Khan

**Affiliations:** ^1^Physiologie de la Nutrition and Toxicologie, UMR U866 Institut National de la Santé et de la Recherche Médicale/Université de Bourgogne-Franche Compté/Agro-SupDijon, France; ^2^UMR U866 Institut National de la Santé et de la Recherche Médicale/Université de Bourgogne-Franche Compté, Chimiothérapie et Réponse Anti-tumoraleDijon, France; ^3^Département de Biochimie, Biologie Cellulaire & Moléculaire, Université de Constantine 1Constantine, Alegria

**Keywords:** taste modalities, cancer, inflammation, metabolism, treatment

## Abstract

The sense of taste is responsible for the detection and ingestion of food to cover energetic requirements in health and disease. The change in taste perception might lead to malnutrition that is usually one of the frequent causes of morbidity and mortality in patients with cancer. In this review, we summarize the mechanisms of taste perception and how they are altered in cancer. We also address the question of the implication of inflammation, responsible for the alterations in taste modalities. We highlight the role of radio- and chemotherapy in the modulation of taste physiology. Other several factors like damage to taste progenitor cells and disruption of gut microbiota are also dealt with relation to taste perception in cancer. We further shed light on how to restore taste acuity, by using different preventive methods, dietary modifications and pharmacotherapy in subjects with advanced cancer state.

## Introduction

The dietary habits are governed, in part, by oro-sensory detection of taste. In fact, the taste of nutrients leads human beings to decide quickly to accept or reject a food. Basically, there are five taste modalities, i.e., sweet, sour, bitter, salty, umami and perhaps, a sixth fat taste (Heinze et al. (2015). Beside the fact that taste is essential for life because it regulates food intake, taste also provides hedonic pleasure from eating. The taste perception also activates neuronal pathways, leading to the preparation for digestion, absorption, and storage of nutrients (Brondel et al., 2013). A dysfunction of taste perception (dysgeusia) may impair the quality of life by affecting appetite, body weight, and psychological well-being (Deems et al., 1991). There are several factors that may affect taste perception, including medication, nutrition, lesions in the oral mucosa, prolonged exposure to radiations and chemotherapy, smoking, chronic hepatitis, renal dysfunction, aging, and perturbation in hormonal secretions (Maffeis and Silva-Netto, 1989).

Changes in taste perception are especially important in diseases like cancer, which is one of the main causes of morbidity and mortality throughout the world. Altered taste perception in cancer subjects is usually ignored by clinicians as this aspect does not represent the life-threatening events. There are some indications that taste alteration might be an alarming early sign of tumor cell invasion in cancer patients (Sherry, 2001). Indeed, the most distressing symptom in patients with advanced cancer is gastrointestinal abnormalities, whereas the change in taste is the fourth most common symptom after dry mouth, weight loss, and early satiety (Komurcu et al., 2002). Some studies suggests that 15 to 100 percent of cancer patients may suffer from a taste change (Lockhart and Clark, 1989; Ripamonti et al., 1998).

## Mechanism of taste perception

Before discussing the relationship between cancer and taste changes, we must have an idea of oro-sensory perception of taste. Taste is a complex entity that interacts with other senses: Hearing, touch, smell, and vision. All the information from the sensory organs is finally analyzed by the central nervous system (CNS). During mastication, foods is mixed with saliva which is secreted by mandibular, sublingual, and parotid salivary glands. Saliva dilutes and disseminates palatable molecules to the taste receptors on the tongue, palate, larynx, pharynx, and the upper third of the esophagus (Matsuo, 2000). Taste receptors or taste receptor cells (TRCs) have been identified on tongue epithelium and throughout the digestive tract (Rozengurt and Sternini, 2007), but we will not discuss the latter part in this review article.

**Figure 1:**
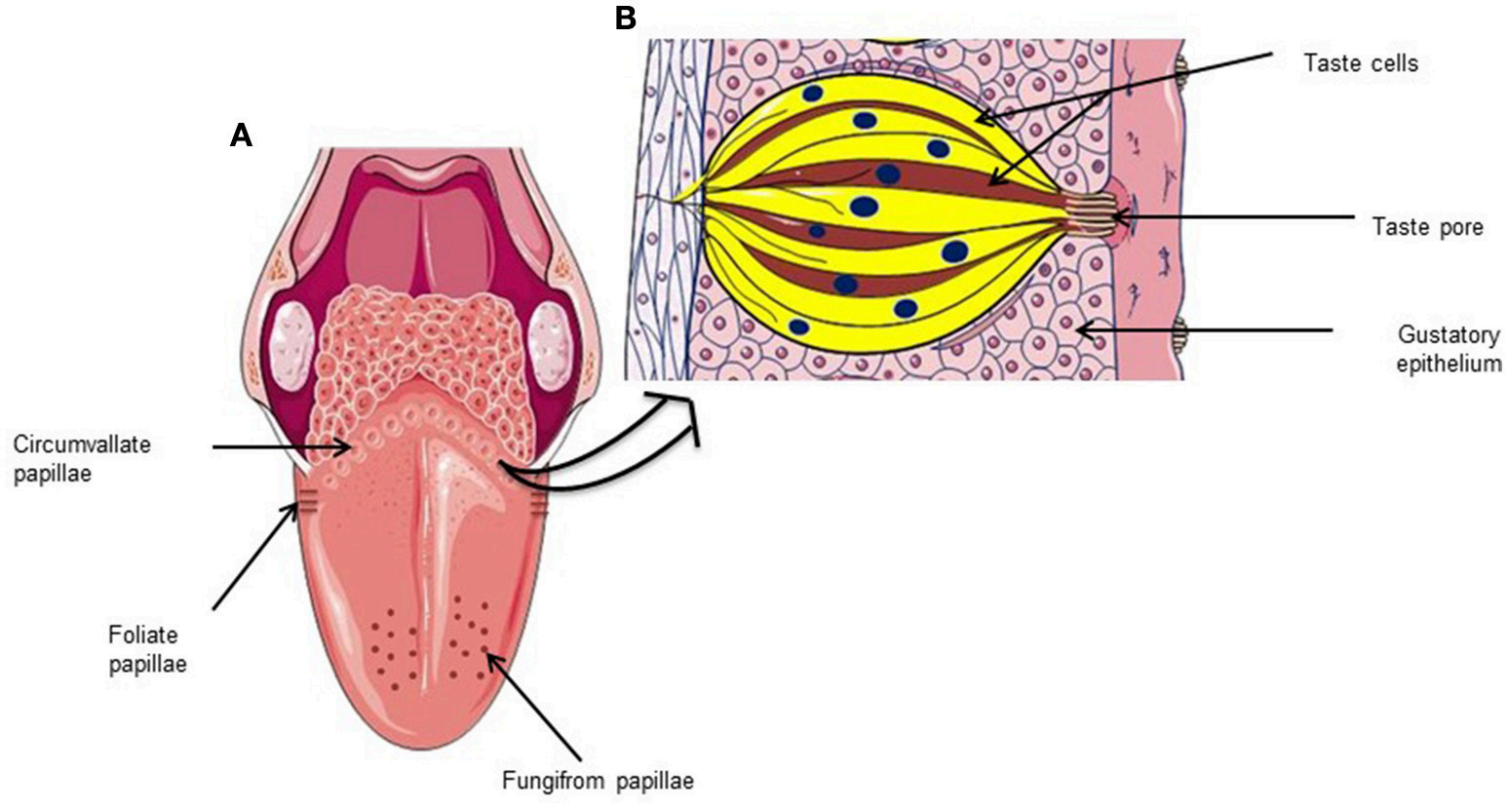
**Human taste system: (A)** Localization of different types of gustatory papillae onto the human tongue. **(B)** Enlarged section of circumvallate papillae showing taste bud cells.

**Figure 2:**
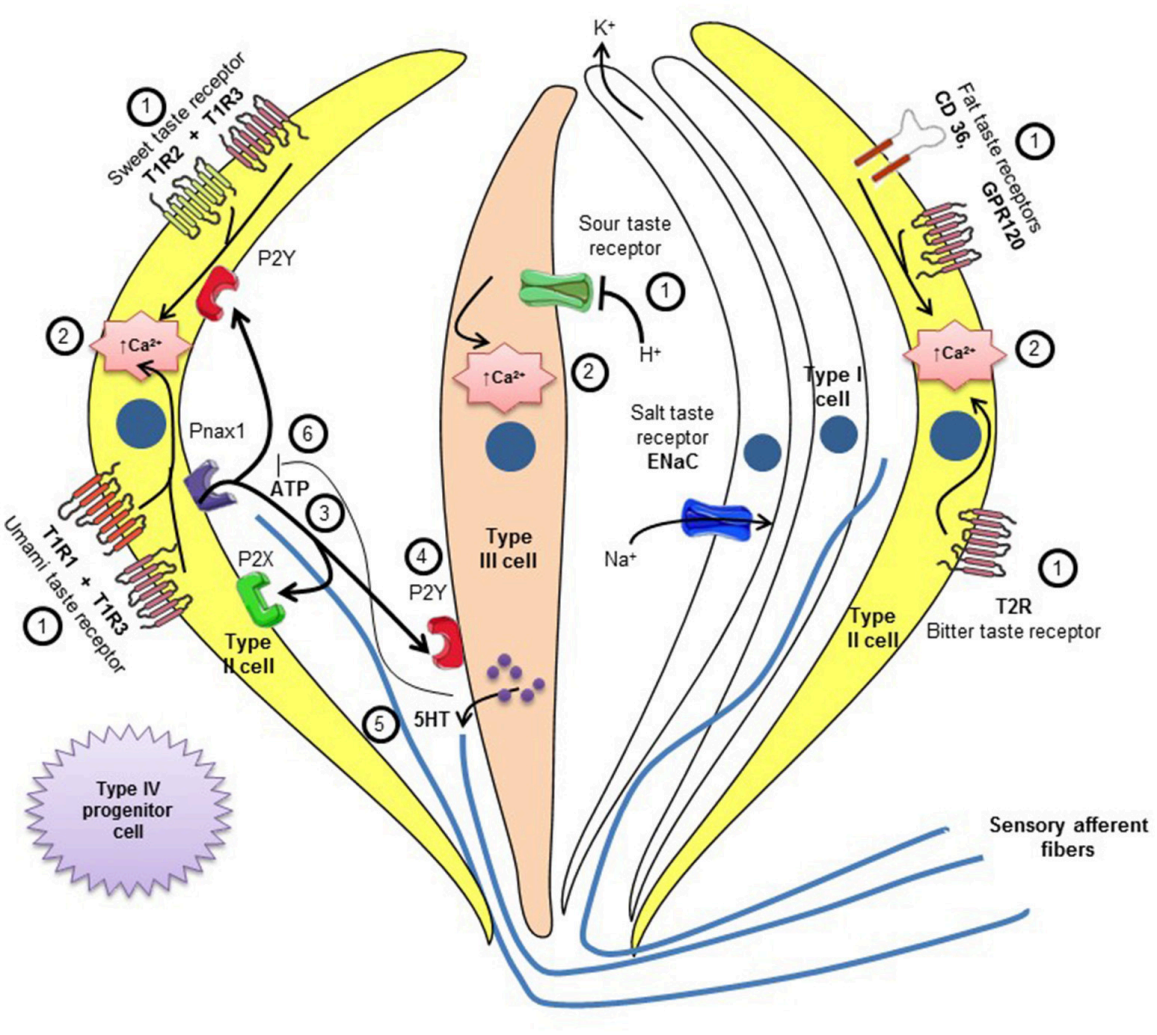
**Intercellular communications involved in taste perception**. Receptors for sweet, umami, fat, and bitter taste are expressed on type-II cells while type III cells express receptors for sour taste and type-I cells are believed to be involved in salt taste perception. Binding of tastants (1) on type-II and type-III cells ultimately leads to an increase in intracellular calcium levels (2). ATP is excreted by type-II(3) cells which binds with P2Y receptors on type-III cells (4) and causes the release of 5-HT (5). 5-HT causes afferent nerve endings to carry taste message to specific brain areas on one side (5), and other the other side, causes feedback inhibition of ATP release (6). Excess ATP is also degrades by ATPase present on type-I cells (not shown). Type IV cells are progenitor cells that differentiate into other three types of cells. (ATP, adenosine triphosphate; 5-HT, 5-hydroxytryptamine or serotonin).

Human beings have around 5,000 taste buds. Of these, 30% are in the fungiform papillae, 30% in foliate papillae and 40% in circumvallate papilla (Suzuki, 2007). The filiform papillae contain no taste buds. Goblet or circumvallate buds are located in the posterior position of the tongue forming an inverted “V” which are nine in humans. The foliate papillae are located in posterior lateral position of the tongue. The fungiform and filiform papillae are present on the apical surface of the tongue. Each taste bud contains 50–100 taste cells, surrounded by supporting cells which are renewed after every 10 days (Wakisaka, 2005). The localization of taste buds and TRCs on human tongue are shown in Figure 1. Three cell types have been identified, based on morphological criteria (Takeda and Hoshino, 1975). Glial or Type I cells that assure homeostasis in the taste bud, are sensitive to salty substances and possess ATPase activity (Bartel et al., 2006), which is crucial to degrade high concentrations of extracellular ATP. The TRCs or type II cells are sensitive to sweet, bitter and umami substances via the receptor activation of T1R and T2R family. These cells secrete ATP in the interstitial medium through pannexin channel (Romanov et al., 2008). The ATP binds to the P2Y receptors on the presynaptic or type III cells. This mechanism results in the release of serotonin, thus activating the postsynaptic receptors which are involved in the transmission of the taste information to brain. The type III cells are sensitive to acidic substances (Yang et al., 2000; Brondel et al., 2013; Roland and and Rémi, 2013). The fourth type of taste cells are termed as type IV cells which are basal, non-polarized, presumably undifferentiated cells and serve as progenitor cells for other three types of taste cells (Miura et al., 2006). The comprehensive reviews on different taste modalities and the activation of different brain areas can be consulted elsewhere (Bermúdez-Rattoni, 2004; Chaudhari and Roper, 2010; Roper, 2013; Besnard et al., 2016). However, in brief, we would like to outline the implication of TRCs in different taste perception. Salt activates sodium channels, while the acidic compounds induce the depolarization via by blocking potassium channels (Kinnamon et al., 1988). Sweet, bitter, fat, and umami taste involve metabotropic receptors (Medler, 2011). CD36 and GPR120 play non-overlapping roles during orosensory detection of dietary fats (Ozdener et al., 2014). Two large families of receptors have been identified: T1R and T2R. The T2R receptors are involved in the perception of bitter taste (Chandrashekar et al., 2000). The heterodimeric T1R2/T1R3 detects the sweet taste (Montmayeur et al., 2001), whereas the heterodimeric T1R1/T1R3 is sensitive to umami taste (Chaudhari et al., 2000). The binding of sapid molecules to these taste receptors activates a G-protein, called Gustducin (Wong et al., 1996). The alpha-subunit of gustducin activates the PLCβ_2_and generates inositol-trisphosphate (IP_3_) and diacylglycerol. All these intracellular mechanisms lead to an increase in intracellular calcium and neurotransmitter release. A diagrammatical representation of various types of taste cells and receptors involved in taste perception are presented in Figure 2. The neurotransmitters released by the TRC activate the afferent nerve fibers that carry taste information to the CNS. These afferent nerves are: (i) the cord of the eardrum (gustatory branch of the facial nerve) which connects the fungiform papillae of the anterior two thirds of the tongue, (ii) the glossopharyngeal nerve (IX) that connects the goblet buds in the posterior third, and (iii) the superior laryngeal vagus nerve (nerve X) that transmits oropharyngeal sensitivity. Other nerves, such as the trigeminal nerve, are also incidentally involved in taste perception. Neurons conduct a first relay in the solitary nucleus, located in the dorsolateral part of the bulb, and then make a second relay in the ventromedial nucleus of the thalamus before they project into the cortical or other area, involved in identifying taste (type and intensity). The nucleus accumbens and the ventral tegmental area are also activated following food ingestion. They are involved in hedonic responses (pleasure) and the reward circuit (Kettaneh et al., 2002; Brondel et al., 2013; Roland and and Rémi, 2013).

## Cancer: inflammation and taste alterations

One of the striking features of advanced cancer is the inflammatory state that is generally associated with an infection (Coussens and Werb, 2002). Rapidly proliferating cancer cells release a number of cytokines/chemokines which favor the recruitment of macrophages and neutrophils, which, in turn, produce a series of cytokines and cytotoxic mediators including prostaglandins (Kuper et al., 2000). It is mention worthy that 15% of cancer are associated with an infection that might trigger a sustained inflammatory state (Kuper et al., 2000). In neck cancer, the level of pro-inflammatory cytokines, i.e., IL-1α, RANTES, MIG, G-CSF, GM-CSF, INF-γ, TNF-a, IL-17, IL-4, IL-6, and IL-10, in the body is increased by several fold (Johnson et al., 2014). The cytokines like IL-12 and IFNγ have an anti-tumor role, while the cytokines like IL-6, IL-17, and IL-23 are pro-tumor (Lin and Karin, 2007). The cytokines IL-6 and IL-10 are associated with poor prognosis in all types of cancer (Lippitz, 2013). Inflammation plays a crucial role in cachexia (Epstein and Barasch, 2010). Conversely, cancer cachexia is associated with an increase in blood levels of C-reactive protein, cytokines (interleukin 1b, interleukin-6, TNF-α, and leukemia inhibitor factor, LIF) and other tumor derived factors like lipid mobilizing factor (LMF) and protein mobilizing factor (PMF). The LMF and PMF can directly mobilize fatty acids and amino acids, respectively, from adipose tissue and skeletal muscle. Ming-Hua et al. (2016) have recently confirmed that high concentrations of IL-1b, IL-6, TNF-α, and LMF are directly involved in cancer cachexia. Indeed, LMF initiates ubiquitin-dependent catabolic pathway and contributes to weight loss during cancer cachexia (Dimitriu et al., 2005) which is, somehow, associated with loss of taste perception as demonstrated by Maschke et al. (2017). The inflammatory markers via blood circulation may also exert their action in the brain and modulate the areas involved in the control of feeding behavior including smell and taste perception (Argilés et al., 2014). These observations suggest that alteration in taste in cancer patients might be controlled both at taste bud and brain levels. As regards the association between inflammation and taste bud dysfunction that might result into altered taste perception, we can cite the study of Wang et al. (2009). These investigators have reported the expression of Toll-like receptors (TLRs), type I and II interferon (IFN) receptors, and their downstream signaling components in taste tissue. Some TLRs appear to be selectively or more abundantly expressed in taste buds than in non-gustatory lingual epithelium. Immunohistochemical observations have confirmed the presence of these receptor proteins in taste bud cells, of which TLRs 2, 3, and 4 are expressed in type II cells. Administration of TLR ligands and lipopolysaccharides activated IFN-g signaling pathways, up-regulated the expression of IFN-g-inducible genes, and down-regulated the expression of c-fos in taste buds. Interestingly, systemic administration of IFNs triggered apoptosis of taste bud cells in mice and, consequently, contributed to the development of taste disorders. It has been shown that MRL/lpr mice have a high concentration of IFNγ and TNF-a, and INFγ would induce the apoptosis of TRCs. The perception of bitter, sweet and umami taste is also reduced in these mice. This alteration of taste perception is associated with a decrease in the number of gustducin positive cells and renewal of TRCs (Kim et al., 2012).

There also seems a relationship between inflammation and hyperglycemia in cancer since carbohydrate metabolism is altered in cancer patients (Duan et al., 2014), as is the case of type 2 diabetes. The high glucose metabolism in cancer cells might be due to high expression of glucose transporters (Walenta et al., 2000; Hauptmann et al., 2005). Gondivkar et al. (2009) have reported that the subjects with type 2 diabetes may also suffer from alterations in taste thresholds for different taste modalities. Hence, it is possible that the alteration in carbohydrate metabolism and taste perception may be a common mechanism between type 2 diabetes and cancer. However, this statement requires further studies in future.

## Modification of microbiota and taste alterations in cancer

Roughly, there are ~10^14^ microbes residing in human intestine with genetic content almost 100 times higher than that of the human genome (Ley et al., 2006). These microbiota are present throughout the gastrointestinal tract, starting from mouth till the terminal part of large intestine (Rozengurt and Sternini, 2007). Schmidt et al. (2014) have shown that the abundance of oral microbiota was varied in individuals during oral cancer. In another study, it was observed that some bacterial species in the buccal cavity of patients with oral squamous cell carcinoma differed from that of normal volunteers (Mager et al., 2005). Interestingly, the chnages in gut microbiota are closely related with alterations in taste preference in mice (Duca et al., 2012; Swartz et al., 2012). One possible mechanism might be that the modifications of gut microbiota might influence the expression of gut G-protein coupled receptors (Rousseaux et al., 2007), beside manipulating host feeding behavior through hormonal and neural mechanisms (Alcock et al., 2014). Oral mucositis, which is closely related with alterations in taste, is frequently reported in cancer patients undergoing chemotherapy (Knox et al., 2000). Wang et al. (2015) have proposed that disruption of oral microbiota could result in chemotherapy-induced inflammation through toll-like receptors (TLRs) and nucleotide oligomerization domain (NOD)-like receptors (NLRs). Ligands for these receptors like peptidoglycan, lipopolysaccharide, bacterial DNA, and protein flagellin are frequently provided by disrupted microbiota, which results in induction of inflammatory process. As cancer therapy is frequently associated with disrupted microbiota, while alterations of microbiota leads to inflammation and changes is taste perception, it would be natural to assume that disruption of gut microbiota might result in taste changes in cancer patients. However, this argument needs to be studied in future.

## Cancer therapy and changes in taste

### Chemotherapy

Chemotherapy may affect the taste perception (Berteretche et al., 2004; Rehwaldt et al., 2009). In a study conducted on cancer patients undergoing chemotherapy, the prevalence of taste alterations was reported to be as high as 69.9%, and a significant association was found between taste alterations and a change in patient's quality of life such as appetite and fatigue (Zabernigg et al., 2010). In a study, performed on pediatric patients, undergoing chemotherapy, the altered taste perception caused problems in feeding behavior (Skolin et al., 2006). It was suggested that changes had occurred both in the primary gustatory sense as well as in food perception in these patients. Taste-test showed that these patients had increased thresholds for bitter taste (Skolin et al., 2006). Such changes in taste may be resolved within several months after the completion of chemotherapy (Bernhardson et al., 2007). Zinc is an important micronutrient which also plays a role in the perception of taste. Studies suggest that Zinc depletion is closely related to a change of taste in cancer patients (Heyneman, 1996; Yamagata et al., 2003). A possible mechanism by which the drugs treating cancer could cause Zinc deficiency may involve the binding and chelation of Zinc and other heavy metals by sulfhydryl group in their structures, leading to Zinc depletion and loss of taste (Comeau et al., 2001).

### Radiation therapy

**Figure 3:**
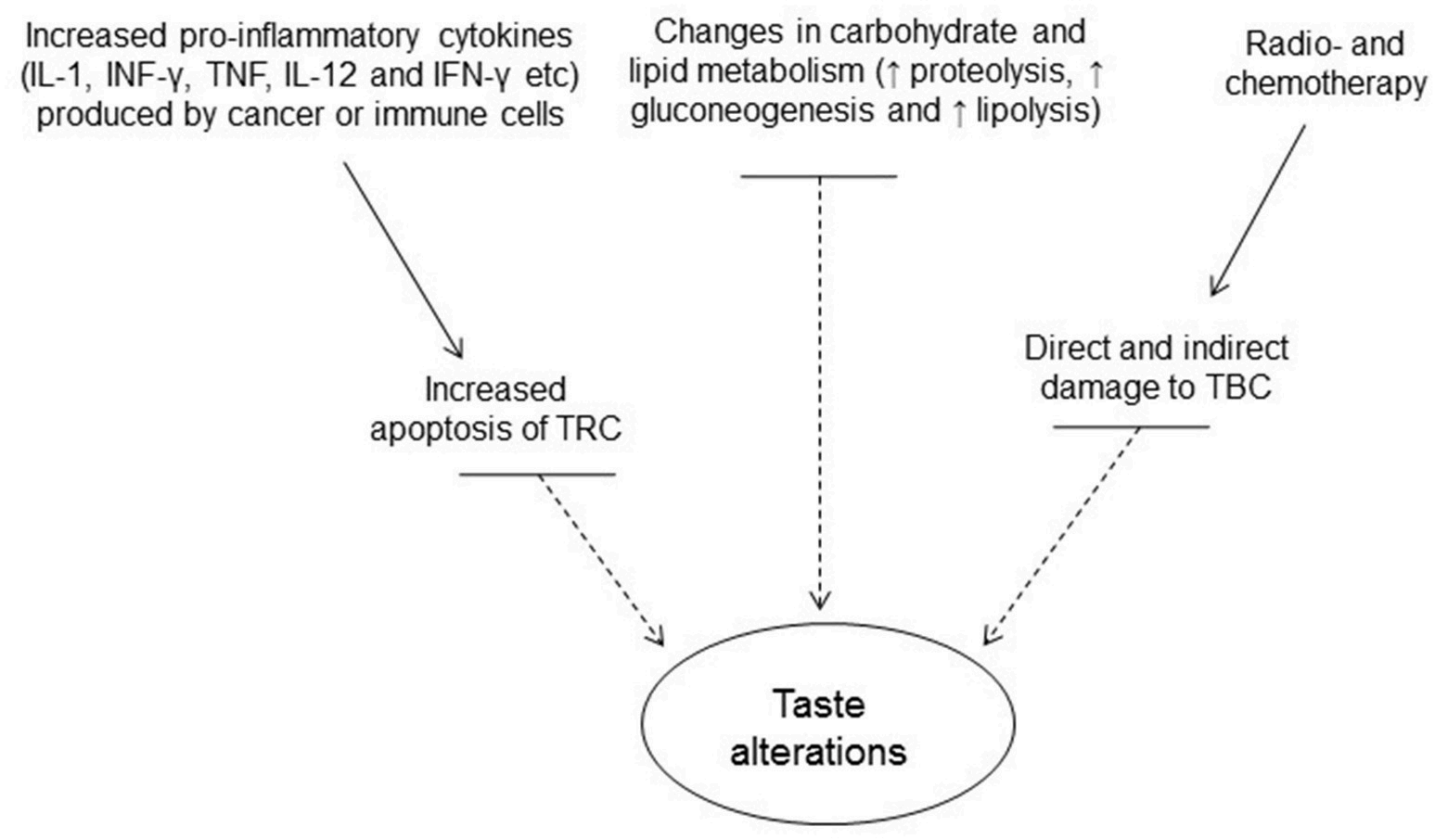
**Factors contributing to alterations in taste perception in cancer**. (TRCs, taste receptor cells; TBCs, taste bud cells).

Taste disorders are quite frequent in patients undergoing radiation therapy for head and neck cancers (Zheng et al., 2002). In a study, it was reported that bitter taste was the most affected taste, while sweet taste was affected to a lesser extent (Zheng et al., 2002). Another study showed that bitter and salty taste were the earliest and the most affected taste modalities, while sweet taste was the least affected as a result of radiation therapy (Mossman and Henkin, 1978). Taste impairment may start a few weeks after the beginning of radiation treatment, while it may recover to its previous levels 6 months to 1 year after the treatment has been stopped but some patients may suffer from permanent loss of taste (Ruo Redda and Allis, 2006). Impairment of ummami taste as a result of radiation therapy in cancer patients has also been reported (Shi et al., 2004). Changes in taste are mainly due to the damage caused by radiation field to taste cells and the pattern of the taste disorder is heavily influenced by the distribution of the taste buds damaged during the radiation therapy (Yamashita et al., 2006). Also, radiation and chemotherapy induce apoptosis of TRCs and inhibit taste progenitor/stem cell proliferation. Role of dry mouth (xerostomia) has also been implicated as a factor contributing to taste change, as radiation therapy frequently affects saliva quantity and composition (Mossman and Henkin, 1978) by damaging salivary glands. A brief summary of the factors that result in taste change has been shown in Figure 3.

### Plausible effect of radio- and chemotherapy on taste progenitor cells

Radiation or chemotherapy might target both taste progenitor as well as already existing taste cells. How these treatments affect differentiation or reduce the cell number by targeting apoptotic or particular pathways are poorly understood. There are pathways known for differentiation of taste bud cells such as sonic hedgehog (SHH) and notch pathways (Kapsimali and Barlow, 2013) which are modulated during cancer (Merchant and Matsui, 2010). In particular, SHH is exclusively expressed in type IV taste cells, which are undifferentiated basal cells and the precursors of the other three types of taste cells (Miura et al., 2006). Use of SHH pathway inhibitor, Vismodegib, in cancer patients is often associated with profound alterations in taste sensation (Von Hoff et al., 2009; Sekulic et al., 2012; Tang et al., 2012). Yang et al. (2015) have shown that Vismodegib treatment in mice resulted in an altered taste perception. Significant reductions in taste bud size, numbers of taste cells per taste bud, and numbers of SHH-expressing cells in taste bud were also observed in these mice. Furthermore, the numbers of phospholipase Cβ2- and α-gustducin- T1R3, glucagon-like peptide-1, and glucagon-expressing cells were also reduced in the taste bud of these mice. Similar findings were observed by Kumari et al. (2015), who reported that the blockade of hedgehog pathway by cancer drug LDE225 resulted in the disruption of taste cells and taste perception in mice. All these findings suggest that modulation of SHH by cancer therapy might also be implicated in taste alterations in cancer patients.

Miura and Barlow (2010) have proposed that in head and neck cancer patients, taste bud progenitors are more vulnerable to the effect of fractionated radiotherapy as compared to terminally differentiated taste cells. They have compared this model with irradiated skin epithelium, where proliferating skin cells are more prone to radiation induced DNA damage than the post-mitotic cells. Following irradiation, the progenitor cells try to repair damaged DNA, and if they fail, they start apoptosis. Repeated irradiation causes increased loss of progenitor cells (Dörr et al., 2000; Potten et al., 2002). As chemotherapy also targets rapidly dividing cells, consequently, taste alterations are observed in patients undergoing chemotherapy for non**-**head/neck cancers as well.

## Measurement of taste impairment and detection threshold in cancer

Different methods have been used for the measurement of taste impairment and detection thresholds in cancer patients. A detailed account of the methods will be out of scope of this article; hence, only a brief summary has been given. An electro-gustometer can be used for the detection of taste thresholds (Williams and Cohen, 1978). One electrode is the tongue electrode, while the other reference electrode is placed on dorsal side or the wrist. Electrical current is applied in different steps and the lowest current intensity, perceived by the subject, is taken as the detection threshold (Berteretche et al., 2004). Chemical detection involves using tastant solution with suprathreshold concentrations for each of the basic taste modalities (Trant et al., 1982; Bossola et al., 2007). The subject's mouth is rinsed with a sip of distilled water prior to testing each sample (Sánchez-Lara et al., 2010). The lowest concentration of solute at which the individual consistently recognizes the taste is considered as taste threshold (Gallagher and Tweedle, 1983; Steinbach et al., 2009). A 3-armed forced choice (3-AFC) method can be applied to monitor the subject's response for detection and reorganization of taste (Mossman and Henkin, 1978). Subjects can be asked to place a cross-hatch on a 10-cm line labeled at each end (0 = dislike extremely, no sweetness, sourness, saltiness, or bitterness, and 10 = like extremely, extremely sweet, sour, salty, or bitter) to indicate degree of liking and intensity of each sample (Trant et al., 1982). Alternatively, a scale ranging from 0 (total taste loss) to 3 (no taste loss) can be employed to classify the subjects, depending upon if they recognize no or all concentrations of a particular taste (Maes et al., 2002). Questionnaires have also been used by different researchers to determine the patient's self-reported taste changes and their effects on their quality of life (Chencharick and Mossman, 1983; Huldij et al., 1986; Bjordal et al., 1994; Bernhardson et al., 2008). The choice of a particular test depends on the practitioner.

## Self-care strategies by patients to minimize alterations in taste

The patients themselves can assess the changes in thresholds for different taste modalities. Consequently, they may change either their feeding habits or adopt appropriate palatable strategies. For example, breast cancer patients, undergoing docetaxel or paclitaxel chemotherapy, included a number of strategies like changing food habits by adding new recipes (Speck et al., 2013). Wickham et al. (1999) proposed to increase food palatability by adding artificial falvours. Rehwaldt et al. (2009) have reported that each patient should have its own particular strategy to adapt taste alterations. Hence, more than 50% of the patients reported that they tried selected one of the following strategies: More fats and sauces, eating smaller and more frequent meals, using more condiments, eating blander foods, adding something sweet to meats, sucking on hard candy, eating more boiled foods, and avoiding beef. These strategies were helpful for the majority (74–87%) of patients who tried them. Nonetheless, self-care strategies are difficult to be practized due to psychological constraints.

## Prevention and treatment of taste alterations

Patient counseling can be started in advance to prepare patient mentally before time. Rhodes et al. (1994) have demonstarted if the patients are prepared psychologically for taste alterations, they can tolerate taste changes easily. Ravasco et al. (2003) have stressed on counseling the cancer patients to face the altered changes in taste perception. Before chemotherapy, the patients can be encouraged to try new food products or supplements (Capra et al., 2001). Lemon juice and chewing gum could be used prior to meals to make the meals more pleasant. Small but frequent meals should be encouraged as they are better tolerated by the patients (Ravasco, 2005). Patients can also be asked to maintain good oral hygiene as it may also contribute to changes in taste. To assess whether changes in taste perception in cancer patients are causing taste aversion and, consequently, to malnutrition, the clinicians may use different approaches, like Malnutrition Screening Tool (Ferguson et al., 1999), Interdisciplinary Nutrition Care Plan (Capra et al., 2001), or the Patient-Generated Subjective Global Assessment (Ferguson, 2003). A close contact and relationship between health care professionals and patients is highly desirable in order to assess the nutritional status and improve the quality of life of the patients.

Zinc supplementation can be valuable for patients undergoing cancer chemotherapy. The results of a pilot study suggested that after 2 weeks of chemotherapy, the intake of Zinc protected the cancer patients against taste disorders (Yamagata et al., 2003). In another study, a Zinc containing formulation known as Polaprezinc was able to improve the taste alterations in 70 percent of the patients, though an early administration has been suggested (Mizukami et al., 2016). Furthermore, the results of a randomized and controlled clinical trial on the effects of Zinc sulfate have clearly demonstrated that exogenous Zinc improved taste acuity (Ripamonti et al., 1998). Similar findings were observed in a randomized placebo-controlled trial where Zinc sulfate prevented the radiation-induced taste changes in head/neck cancer patients (Najafizade et al., 2013). Halyard et al. (2007) conducted a clinical study on Zinc sulfate vs. placebo and concluded that intake of Zinc offered protection against alterations in taste modalities. Hence, the routine use of Zinc sulfate may be suggested for patients undergoing cancer therapy.

Amifostine, an organic thiophosphate, has been shown to protect normal tissues from damage caused by radiation and chemotherapy (Kouvaris et al., 2007). Amifostine may also play a role in the protection of salivary glands and, thus leading to improvement of xerostomia. In a phase II randomized trial, amifostine has been used to assess protection against the toxic effects of carboplatin (Bohuslavizki et al., 1998). Münter et al. (2007) also came to the similar conclusion that amifostine may prevent reduced salivary gland function in patients, subjected to radiotherapy.

The efficacy of two drugs, i.e., pilocarpine and bethanechol, that increase production of saliva was tested upon saliva secretion in cancer patients with hypo-salivation following radiation therapy (Gorsky et al., 2004). Though the patients reported improvement in saliva secretion by both the agents, only bethanechol improved taste perception.

A pilot investigation was conducted to assess the role of serotonergic blockade by using ondansetron, an antagonist of serotonin type-3 receptor (Edelman et al., 1999). This study included metastatic cancer patients who were not undergoing chemotherapy or radiotherapy. Utilizing an “extensive battery of taste tests,” it was concluded that ondansetron brought about significant improvements in patients to enjoy food. A major drawback in this study was that it was not a blinded trial, and, hence, placebo effects could not be fully excluded (Edelman et al., 1999). Moreover, it was not clear whether the enhanced enjoyment from eating was due to improvements in taste. In rodent model, it seems that 5-HT acts as a paracrine inhibitory feedback signal manifested by the inhibition of ATP secretion from TRCs. Taste buds express 5-HT_1*A*_ receptor and the use of its specific agonist, 8-OH-DPAT, inhibited taste-evoked Ca^2+^ release in TRCs and also curtailed ATP release. Conversely, blocking the action of endogenous 5-HT in taste bud cells by using WAY100635, a selective 5-HT_1*A*_ receptor antagonist, increased taste-evoked ATP release (Huang et al., 2009). However, in-depth studies aiming to reproduce the afore-mentioned observations in humans are awaited.

## Future prospects

The pathophysiology of taste alterations differs with relation to the phase of the disease and specific treatment. Unfortunately, this aspect has been ignored in most of the publications. Researchers should take into account the therapy and stages of disease while collecting the data so that more specific patient care protocols can be devised in order to minimize the effect of a change in taste on patient's quality of life. Manufacturers must take into account the taste alterations that frequently accompany cancer and should design and provide food supplements accordingly to make them more palatable. An interesting idea can be to develop “taste enhancers” which, once added to food, could enhance its palatability by acting as agonist at specific taste receptor. Such agonists can be developed for each taste modality. After identification of particular taste impairment, the enhancers for that taste can be added to make food more pleasurable for patients.

## Author contributions

NK and FG proposed the topic, supervised and finally reviewed. BM, AH, and AK participated equally in searching the data and writing of the review.

### Conflict of interest statement

The authors declare that the research was conducted in the absence of any commercial or financial relationships that could be construed as a potential conflict of interest.
